# What's in the Chinese Babyface? Cultural Differences in Understanding the Babyface

**DOI:** 10.3389/fpsyg.2016.00819

**Published:** 2016-05-31

**Authors:** Wenwen Zheng, Qian Yang, Kaiping Peng, Feng Yu

**Affiliations:** ^1^Department of Psychology, Tsinghua UniversityBeijing, China; ^2^Department of Public Health, Zhejiang UniversityHangzhou, China; ^3^Department of Leadership and Organization Management, School of Economics and Management, Tsinghua UniversityBeijing, China

**Keywords:** babyface, cultural differences, facial structure, face perception, trait impressions

## Abstract

We investigated the cultural differences in understanding and reacting to the babyface in an effort to identify both cultural and gender biases in the universal hypothesis that the babyfaced individuals are perceived as naïve, cute, innocent, and more trustworthy. Sixty-six Chinese and Sixty-six American participants were required to evaluate Chinese faces selected from the Chinese Academy of Sciences (CAS)—Pose, Expression, Accessories, and Lighting (PEAL) Large-Scale Chinese Face Database. In our study, we applied Active Shape Models, a modern technique of machine learning to measure facial features. We found some cultural similarities and also found that a Chinese babyface has bigger eyes, higher eyebrows, a smaller chin, and greater WHR (Facial width-to-height ratio), and looks more attractive and warmer. New findings demonstrate that Chinese babyfaces have a lower forehead and closer pupil distance (PD). We found that when evaluating the babyfacedness of a face, Chinese are more concerned with the combination of all facial features and American are more sensitive to specific highlighted babyfaced features. The Chinese babyface tended to be perceived as more babyfaced for American participants, but not less competent for Chinese participants.

## Introduction

You can never tell a book by its cover, but we always automatically and unconsciously judge people by their faces. Indeed, in fewer than 50 ms, first impressions about a face can be generated (Todorov et al., 2009). The babyface, with its unique facial structures which differ from mature adult faces, evokes a series of stereotypes. Whether in humans or animals, the babyface is usually defined as a round face with big eyes, high raised eyebrows, a narrow chin, and a small nose. All these features give us the impression of child-like traits, such as being naïve, cute, innocent, etc. (Zebrowitz and Montepare, 1992, 2005; Zebrowitz et al., 1993, 2012). The babyface overgeneralization effect applies to both infants and adults, including youth and seniors (Zebrowitz and Franklin, 2014; Zebrowitz et al., 2015). Babyface stereotypes can bias social life outcomes, including elections, financial rewards, job applications, academic performances, prison sentences, altruism, and communication environments (Zebrowitz and McDonald, 1991; Zebrowitz et al., 1992, 1998; Collins and Zebrowitz, 1995; Zebrowitz and Montepare, 2008; Livingston and Pearce, 2009; Poutvaara et al., 2009).

There are a few cross-cultural investigations of babyface phenomena in different cultural contexts, but they are focused more on identifying similarities than differences (Zebrowitz et al., 1992, 1993). Zebrowitz et al. (2012) proposed a common mechanism among people's social perception of faces. Even individuals who are isolated from the industrial revolution and modernization can still develop the ability to perceive attractive faces and babyfaces.

However, we believe there is still room for cultural differences in the definition of the babyface and in inferences regarding the babyface in different cultural contexts. The babyface distributes in a given ratio among different ethnic groups, but the definition of the babyface in terms of facial structures and social perceptions varies across cultures. We make judgments about faces based on visual information, cognitive learning such as attention training, and the usefulness of information to observers (Zebrowitz et al., 2011). All of these cognitive strategies have been found to have systematic cultural differences by cultural psychologists for decades (Peng and Nisbett, 1999; Nisbett et al., 2001). Hence, we cannot conclude that babyface perceptions and inferences can easily escape cultural influence from a cultural psychology perspective. It is widely known that the Chinese holistic cognitive style is concerned more with integrated features, and the American analytical cognitive style is concerned more with prominent features. Evidence also suggests that Japanese use more configuration information in face perception than Caucasian (Miyamoto et al., 2011). Therefore, we assumed that Chinese and American participants would differ in babyface perceptions and inferences. Chinese should focus more on the combination of all facial features and Americans should be more sensitive to some highlighted babyfaced features. Forehead height and WHR should show cultural differences, because they are closely related to the integrated perception of a face. Eyebrow height and chin width should show cultural differences, because they are highlighted babyfaced features.

Child-like traits from the babyface have been reported by previous studies. We summarized 17 traits which can be inferred from the babyface, including naïveté, attractiveness, likeability, caring, friendliness, kindness, honesty, trustworthiness, health, openness, extroversion, emotional stability, confidence, intelligence, leadership ability, aggressiveness, and threat (Berry and McArthur, 1985; McArthur and Berry, 1987; Zebrowitz et al., 1992, 1993, 2007b, 2015; Albright et al., 1997; Zebrowitz and Montepare, 2005; Zebrowitz, 2006). These 17 traits were evaluated in our study in researching cultural differences in inferences made about the babyface. A stereotype content model (Caprariello et al., 2009; Cuddy et al., 2009) applies a method to refine these traits. They could be divided into two categories—warmth and competence. We anticipated that the babyface overgeneralization hypothesis and the halo effect of attractiveness should also work on Chinese faces. There should be a positive correlation between attractiveness, health, warm traits and babyfacedness. However, cultural differences may appear when perceiving the trait of competence. There may be no negative correlation between these traits and babyfacedness, because there exists an evolutionary mechanism by which the babyface is a wise strategy to help Chinese people gain more resources. A round face shape, especially a babyface, will help them to get limited resources in limited time, which may increase, not decrease their competence inferences (Buss, 1989; Cunningham et al., 1995). Unlike previous studies, we hypothesized that the Chinese babyface may lead to differences in competence inferences.

## Materials and methods

### Participants

Seventy-two undergraduate students from Tsinghua University, 38 males, and 34 females, participated in the experiment. Data from six participants were excluded from further analyses because they are not Chinese. The valid data included 66 people (32 females, age: *M* ± *SD*, 21.5 ± 3.17 years old; 34 males, 21.56 ± 3.60 years old, range: 18–31 years old). Seventy-eight undergraduate students from the University of California Berkeley, 26 males, 52 females, participated in the experiment. Data from twelve participants who are not Caucasian were excluded from further analyses. The valid data included 66 people (44 females, 21.07 ± 4.89 years old; 22 males, 22.91 ± 4.36 years old, range: 18–34 years old). The study was approved by both Tsinghua University and University of California Berkeley Institutional Review Board, and all participants gave informed consent.

### Stimuli

In this study, Chinese faces were researched as experiment material after being filtered and measured. Chinese faces came from the Chinese Academy of Sciences (CAS)—Pose, Expression, Accessories, and Lighting (PEAL) Large-Scale Chinese Face Database, including 1040 adult volunteers (445 women) (Gao et al., 2008). In the pre-experiment, the black-white photo group with the unified background, light, focal length, neutral expression, and no ornaments was chosen.

**Figure 1:**
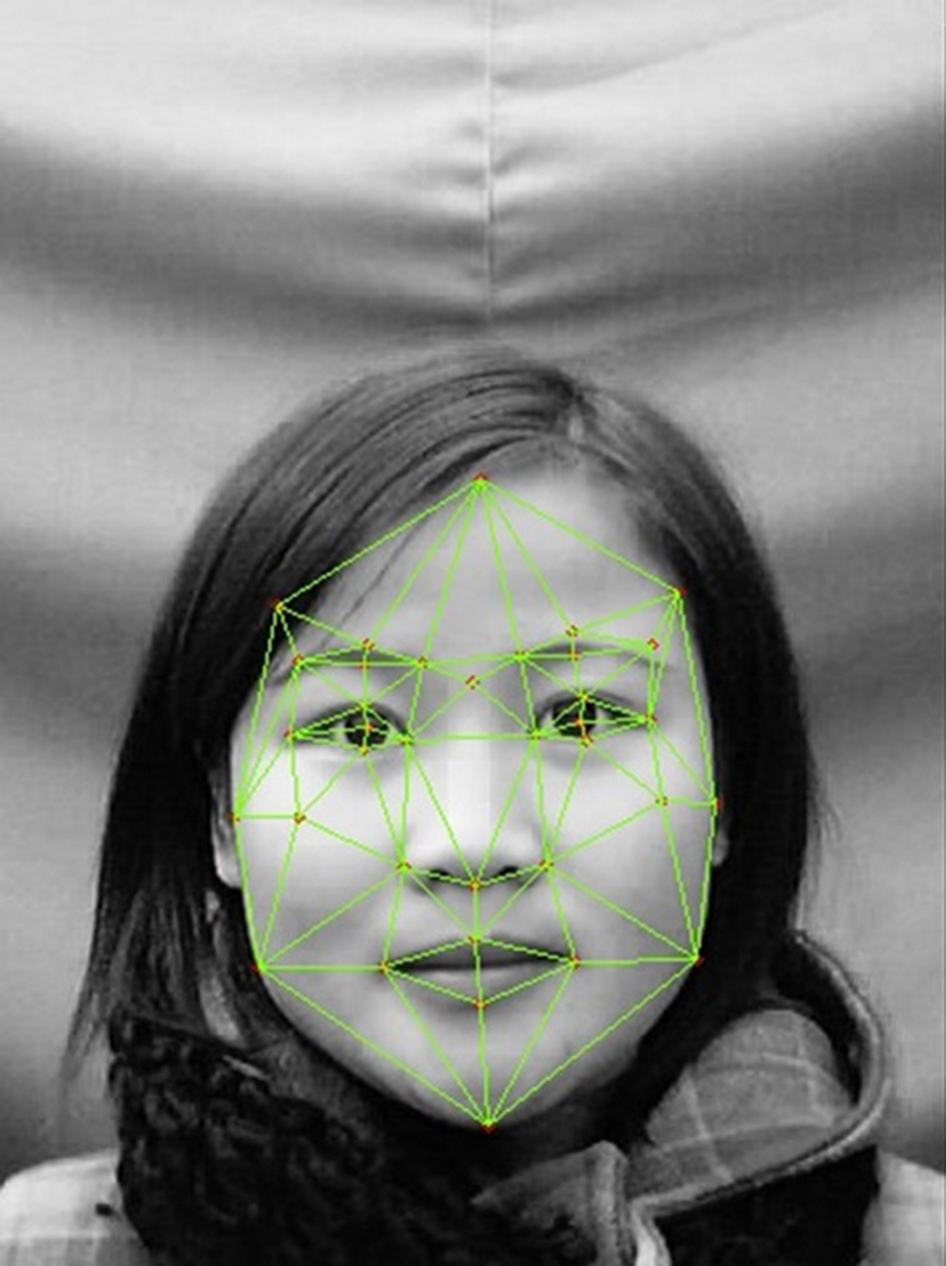
**An example of a pointing picture**.

We applied Active Shape Models to value the level of babyfacedness and ranked all faces in Stasm Software (Milborrow and Nicolls, 2014). All the 1040 faces were first marked with 38 fixed points (Zebrowitz-McArthur and Montepare, 1989; Zebrowitz et al., 2003, 2007b, 2010). The result of photo pointing is to get the coordinate value of 38 fixed points by setting up a rectangular coordinate system with the bottom left point of the screen as the origin (Figure 1). Referring to previous research, 18 feature vectors were described by the coordinates of facial standard points (Table 1). All the values were standardized by pupil distance (PD).

**Table 1:** **Facial Structure Feature Vectors and Fixed Points**.

**No**.	**Feature vector**	**Starting point**	**Terminal point**
1	Forehead height	Highest point of forehead	Highest point of nose
2	Nose height	Highest point of nose	Lowest point of nose
3	Mouth height	Highest point of lips	Lowest point of lips
4	Chin height	Lowest point of lips	Lowest point of chin
5	Left eye height	Highest point of left eye	Lowest point of left eye
6	Right eye height	Highest point of right eye	Lowest point of right eye
7	Forehead width	Rightmost point of forehead	Leftmost point of forehead
8	Left eyebrow height	Medial lowest point of left eyebrow	Center of left pupil
9	Right eyebrow height	Medial lowest point of right eyebrow	Center of right pupil
10	Left eye length	Leftmost point of left eye	Rightmost point of left eye
11	Right eye length	Leftmost point of right eye	Rightmost point of right eye
12	Face width	Right zygoma point	Left zygoma point
13	Nose width	Lateral point of right ala nasi	Lateral point of Left ala nasi
14	Mouth width	Rightmost point of lips	Leftmost point of lips
15	PD	Center of right pupil	Center of left pupil
16	Face height	Highest point of forehead	Lowest point of chin
17	Left cheek smoothness degree (the radius of a circle of the triangle combined by three lines)	Left ear	Lowest point of chin
		Left mandible	Lowest point of chin
		Left ear	Left mandible
18	Right cheek smoothness degree (the radius of a circle of the triangle combined by three lines)	Right ear	Lowest point of chin
		Right mandible	Lowest point of chin
		Right ear	Right mandible

Following Berry and McArthur (1985) and Zebrowitz-McArthur and Montepare (1989), we found that the feature vectors significantly correlated with the babyfacedness and the correlation coefficients between them (Table 2). The standardized vectors, which were selected, were eye size, eyebrow height, forehead height (from highest point of forehead and highest point of nose), eye shape, chin width, PD, and cheek smoothness degree.

**Table 2:** **Selected vectors and correlation coefficients with babyfacedness**.

**No**.	**Feature vector**	**Correlation coefficients**
r_1_	Eye size	0.40
r_1_'	Eye size	0.48
r_2_	Eyebrow height	0.43
r_3_	Forehead height	0.46
r_4_	Eye shape	0.51
r_5_	Chin width	0.61
r_6_	PD	0.66
r_7_	Cheek smoothness degree	0.48

r_1_ is from Berry and McArthur (1985). r_1_' is from Zebrowitz-McArthur and Montepare (1989). r_2_ is from Berry and McArthur (1985). r_3_ is from Zebrowitz-McArthur and Montepare (1989). r_4_ is from Berry and McArthur (1985). r_5_ is from Berry and McArthur (1985). r_6_ is from Zebrowitz-McArthur and Montepare (1989). r_7_ is from Zebrowitz-McArthur and Montepare (1989).

Initial filter formula as follows, DA, DB, and DC represent the value of babyfacedness.

(1) $$\begin{array}{l} DA\;=\;Eye\;size\;+\;Eyebrow\;height\;+\;{Forehead\;height}_{1}\\ +\;Chin\;width\;+\;PD\;+\;Cheek\;smoothness\;degree \end{array}$$

(2) $$\begin{array}{l} DB\;=\;r_{1}\;\times \;Eye\;size\;+\;r_{2}\;\times \;Eyebrow\;height\;+r_{3}\\ \times \;{Forehead\;height}_{2}\;+\;r_{4}\;\times \;Eye\;shape\;+\;r_{5}\\ \times \;Chin\;width\;+\;r_{6}\;\times \;PD\;+\;r_{7}\;\\ \times \;Cheek\;smoothness\;degree \end{array}$$

$$\begin{array}{l} DC\;=\;{r\prime }_{1}\;\times \;Eye\;size\;+\;r_{2}\;\times \;Eyebrow\;height\;+r_{3}\\ \times \;{Forehead\;height}_{2}\;+\;r_{4}\;\times \;Eye\;shape\;+\;r_{5}\\ \times \;Chin\;width\;+\;r_{6}\;\times \;PD\;+\;r_{7}\;\\ \times \;Cheek\;smoothness\;degree \end{array}$$

Comparing the three kinds of babyfacedness value, significant positive correlations were found between DA and DB, *r*_(46)_ = 0.96, *p* < 0.01; DA and DC, *r*_(46)_ = 0.96, *p* < 0.01; DB and DC, *r*_(46)_ = 1.00, *p* < 0.01. It can be inferred that the difference of correlation coefficients had little influence on babyfacedness value. Faces were ranked by the average value of DA, DB, and DC.

In the list of faces, 23 male faces and 23 female faces were chosen as the stimuli where the step size equaled to 20, from the least babyfaced to the most babyfaced. For each level, the babyfacedness values of faces are close. We controlled three confounding variables in our study: First, age. The faces are between the ages of 22 and 45 as inferred from their appearance (23 females, 29.14 ± 4.87 years old; 23 males, 30.46 ± 5.44 years old). Second, attractiveness. The face which was chosen is the face with average attractiveness among faces of each level. Third, front bangs. We chose the faces in which the forehead can be seen, instead of those in which the forehead is totally covered by front bangs. By controlling these confounding variables, we can not only control the unexpected influence of attractiveness, perceived age and hair style, but also avoid the confound effects of the attractiveness of faces on levels of babyfacedness.

### Procedure

The experiment was conducted in a quiet and bright lab. Participants were asked to fill in the questionnaire individually on a computer with 17 inch LCD monitor (1280 × 1024, 60 HZ). All face images were displayed in 360 × 480 pixel size, 96-dpi. After studying the concept of the babyface: “Babyface, referring to the facial features of those with newborns face,” participants were asked to practice choosing a more babyfaced face from two female and two male faces. If the choice was not correct, a second trial was conducted. If the choice was still wrong at the second trial, then participants were sent back to study the concept of the babyface. They would not enter the next trial until the choice was right.

The gender of faces was randomly presented in the formal trial to ensure the same presentation times of female and male faces. Everyone should react to only one gender. There was no right or wrong in the formal experiment.

The first stage was to practice a forced choice task. 23 faces were presented randomly in pairs, totaling 253 pairs. Participants were asked to judge the two faces with the same gender (two male faces or two female faces), and then give a reaction as soon and as correctly as possible. They pressed S, if the left face was more babyfaced. And L, if the right one was more babyfaced. There was no time limit. The purpose of forced choice task is to make participants practice their definition of babyface and decide what makes a face look babyfaced. The results were not considered in our final analysis or evaluation.

Second, participants were asked to grade the babyfacedness of 23 faces (male or female) presented randomly from 0 to 100, with 0 = the least babyfaced, and 100 = the most babyfaced.

In the rest stage, participants could rest for 10 minutes.

Third, according to the grade of every face given by the participant in the second stage, the most babyfaced and the least babyfaced faces were presented randomly. On a 7-point Likert-scale, participants evaluated 17 traits, including naïveté, attractiveness, likeability, caring, friendliness, kindness, honesty, trustworthiness, health, openness, extroversion, emotional stability, confidence, intelligence, leadership ability, aggressiveness, and threat. And last, participants graded the babyfacedness of these two faces again from 0 to 100 and answered how certain they were of their judgment.

When participants finished all three stages, the experiment was over. American participants used the same program as the Chinese, while the language was English. The introduction was translated by a bilingual speaker and checked by a native speaker.

## Results

### Facial structures of the babyface

With the Kolmogorov-Smirnov test, we found that the grades of babyfacedness given by Chinese and American participants showed no systematic difference. For female faces, *D* = 0.44, *p* = 0.99. For male faces, *D* = 0.30, *p* = 0.24.

Hierarchical linear modeling (HLM) techniques were used to investigate the effect of facial structures and culture on the perception of babyfacedness. The outcome variable is babyfacedness, which was rated by the evaluation of individual faces on a 100-point scale. The face-level, which is level 1, consists of face gender (a dummy variable, 0 = Female, 1 = Male) and eight facial features (cheek smoothness degree; eye size; forehead height; eyebrow height; chin width; eye separation; PD; Facial width-to-height ratio, WHR). The culture-level, which is level 2, consists of two cultural backgrounds, both Chinese and American. Culture is a dummy variable (0 = Chinese; 1 = American).

Table 3 presents descriptive statistics and correlations among the variables. Table 4 shows the HLM results. As shown in Model 1 of Table 4, face gender (γ = 4.69, *p* = 0.02), eye size (γ = 227.41, *p* < 0.01), eyebrow height (γ = 240.98, *p* < 0.01) and WHR (γ = 160.51, *p* < 0.01) are positively related to babyfacedness, but forehead height (γ = −26.69, *p* < 0.01), chin width (γ = −31.03, *p* < 0.01), and PD (γ = −0.50, *p* < 0.01) are negatively related to babyfacedness.

**Table 3:** **Means, standard deviations, and correlations between the variables**.

**Level**	**1**	**2**	**3**	**4**	**5**	**6**	**7**	**8**	**9**
**FACE/LEVEL-1 (*N* = 3036)**									
Babyfacedness	(0.85)								
Cheek smoothness degree	−0.08^**^, *p* < 0.01								
Eye size	0.01, *p* = 0.66	−0.26^**^, *p* < 0.01							
Forehead height	0.05^**^, *p* = 0.01	−0.11^**^, *p* < 0.01	0.28^**^, *p* < 0.01						
Eyebrow height	0.00, *p* = 0.98	−0.13^**^, *p* < 0.01	0.05^**^, *p* < 0.01	−0.47^**^, *p* < 0.01					
Chin width	0.01, *p* = 0.51	−0.24, *p* < 0.01	0.18^**^, *p* < 0.01	−0.08^**^, *p* < 0.01	0.27^**^, *p* < 0.01				
Eye separation	0.08^**^, *p* < 0.01	−0.13^**^, *p* < 0.01	−0.41^**^, *p* < 0.01	−0.17^**^, *p* < 0.01	0.09^**^, *p* < 0.01	0.12^**^, *p* < 0.01			
Pupil distance	−0.19^**^, *p* < 0.01	0.38^**^, *p* < 0.01	−0.28^**^, *p* < 0.01	−0.32^**^, *p* < 0.01	−0.07^**^, *p* < 0.01	−0.31^**^, *p* < 0.01	0.05^**^, *p* = 0.01		
WHR (Facial width-to-height Ratio)	0.25^**^, *p* < 0.01	0.02, *p* = 0.30	−0.27^**^, *p* < 0.01	0.40^**^, *p* < 0.01	−0.62^**^, *p* < 0.01	−0.11^**^, *p* < 0.01	0.26^**^, *p* < 0.01	−0.13^**^, *p* < 0.01	
*M*	49.53	0.97	0.21	0.82	0.29	1.21	0.58	81.80	1.59
*SD*	25.77	0.02	0.02	0.09	0.03	0.08	0.02	6.22	0.07
**CULTURE/LEVEL-2 (*N* = 132)**									
*M*	0.50								
*SD*	0.50								

The face-level, which is level 1, sample consisted of face gender (a dummy variable, 0 = Female, 1 = Male) and eight facial features. The culture-level, which is level 2, sample consisted of two cultural backgrounds, both Chinese and American. Culture is a dummy variable (0 = Chinese; 1 = American). Reliability estimates (coefficient alpha) are in parentheses on the diagonal. Pupil distance is used to standardize the variables of Round, Eye size, Forehead, Eyebrow, Chin, and Eye separation. Significant differences are indicated:

** p < 0.01 (two-tailed tests).

**Table 4:** **Hierarchical Linear Modeling Result: Effect of Facial Structures and Culture on the Babyface**.

**Dependent variable at level 1**	**Babyfacedness**	
	**Model 1**	**Model 2**
**LEVEL-1**		
• Face gender (γ_10_)	4.69^*^ (2.07), *p* = 0.02	5.44 (3.03), *p* = 0.07
• Cheek smoothness degree (γ_20_)	−27.38 (24.15), *p* = 0.26	−14.36 (35.66), *p* = 0.69
• Eye size (γ_30_)	227.41^**^ (31.61), *p* < 0.01	171.00^**^ (43.77), *p* < 0.01
• Forehead height (γ_40_)	−26.69^**^ (5.33), *p* < 0.01	−38.08^**^ (8.50), *p* < 0.01
• Eyebrow height (γ_50_)	240.98^**^ (22.21), *p* < 0.01	176.25^**^ (32.05), *p* < 0.01
• Chin width (γ_60_)	−31.03^**^ (5.45), *p* < 0.01	−20.24^**^ (7.77), *p* = 0.01
• Eye separation (γ_70_)	−17.92 (20.18), *p* = 0.38	−39.58 (29.33), *p* = 0.18
• Pupil distance (γ_80_)	−0.50^**^ (0.07), *p* < 0.01	−0.40^**^ (0.10), *p* < 0.01
• WHR (γ_90_)	160.51^**^ (10.02), *p* < 0.01	136.18^**^ (14.27), *p* < 0.01
**CROSS LEVEL**		
• Intercept (γ_00_)	−185.61^**^ (32.23), *p* < 0.01	−129.45^**^ (45.83), *p* < 0.01
• Culture (γ_01_)		−112.32 (63.77), *p* = 0.08
• Face gender × Culture (γ_11_)		−1.49 (4.14), *p* = 0.72
• Cheek smoothness degree × Culture (γ_21_)		−26.04 (48.25), *p* = 0.59
• Eyesize × Culture (γ_31_)		112.81 (62.08), *p* = 0.07
• Forehead height × Culture (γ_41_)		22.77^*^ (10.44), *p* = 0.03
• Eyebrow height × Culture (γ_51_)		129.46^**^ (42.51), *p* < 0.01
• Chin width × Culture (γ_61_)		−21.59^*^ (10.74), *p* = 0.04
• Eye separation × Culture (γ_71_)		43.32 (39.96), *p* = 0.28
• Pupil distance × Culture (γ_81_)		−0.20 (0.13), *p* = 0.13
WHR × Culture (γ_91_)		48.66^**^ (19.45), *p* = 0.01
• ~*R*^2^	0.16	0.17

Level 1 is the Face Level (N = 3036); Level 2 is the Culture Level (n = 132). Values in parentheses are standard errors. All entries corresponding to the predicting variables are unstandardized estimations of the fixed effects, γ s, with robust standard errors. Significant differences are indicated:

* p < 0.05,

** p < 0.01 (two-tailed tests).

**Figure 2:**
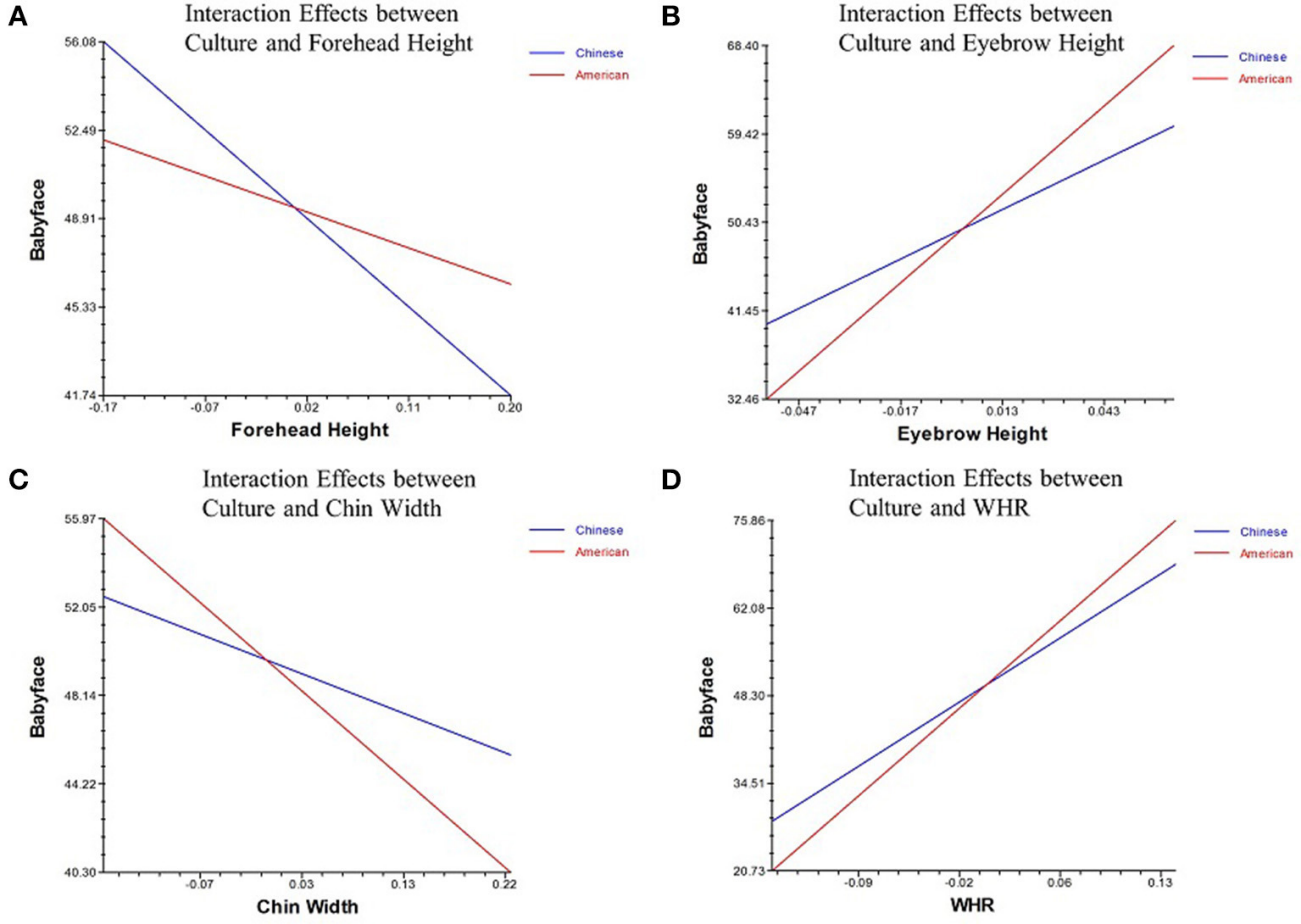
**Interaction Effects among the facial features and culture**. **(A)** Interaction effects between forehead height and culture on babyfacedness. **(B)** Interaction effects between eyebrow height and culture on babyfacedness. **(C)** Interaction effects between chin width and culture on babyfacedness. **(D)** Interaction effects between WHR and culture on babyfacedness.

Examining the effects of culture, Model 2 of Table 4 shows that culture has interaction effects with forehead height (γ = 22.77, *p* = 0.03) (Figure 2A), eyebrow height (γ = 129.46, *p* < 0.01) (Figure 2B), chin width (γ = −21.59, *p* = 0.04) (Figure 2C), and WHR (γ = 48.66, *p* = 0.01) (Figure 2D) on babyfacedness.

The mixed model is:

$$\begin{array}{l} \text{Babyfacedness}=\gamma_{00}+\gamma_{01}{}^{*}Culture+\gamma_{10}{}^{*}Face\;gender+\gamma_{11}{}^{*}Culture^{*}Face\;gender\;+\gamma_{20}{}^{*}Cheek\;smoothness\;degree\\ \;+\;\gamma_{21}{}^{*}Culture{\;}^{*}Cheek\;smoothness\;degree+\gamma_{30}{}^{*}Eye\;size+\gamma_{31}{}^{*}Culture{\;}^{*}Eye\;size+\gamma_{40}{}^{*}Forehead\;height\\ \;+\;\gamma_{41}{}^{*}Culture^{*}Forehead\;height+\gamma_{50}{}^{*}Eyebrow\;height+\gamma_{51}{}^{*}Culture^{*}Eyebrow\;height+\gamma_{60}{}^{*}Chin\;width\\ \;+\;\gamma_{61}{}^{*}Culture^{*}Chin\;width+\gamma_{70}{}^{*}Eye\;separation+\gamma_{71}{}^{*}Culture^{*}Eye\;separation+\gamma_{80}{}^{*}Pupil\;distance\\ \;+\;\gamma_{81}{}^{*}Culture^{*}Pupil\;distance+\gamma_{90}{}^{*}WHR+\gamma_{91}{}^{*}Culture^{*}WHR \end{array}$$

Table 5 shows results of multiple regression analysis predicting babyfacedness from facial structures. The dependent variable is the average of babyfacedness rating of each face. We applied a backward elimination method in our analysis. Results showed facial structures contributed differently to babyface perception in the two cultures. For Chinese judges, WHR, PD, and forehead height contribute more to babyface perception than other cues. For American judges, WHR, PD, and eyebrow height made greater contributions.

**Table 5:** **Results of multiple regression analysis predicting babyfacedness from facial structures**.

**Predictor variable**	**Chinese**				**Predictor variable**	**USA**			
	**Standardized coefficients (Beta)**	***T* (p)**	**95% Confidence interval for B**			**Standardized coefficients (Beta)**	***T* (p)**	**95% Confidence interval for B**	
			**Lower bound**	**Upper bound**				**Lower bound**	**Upper bound**
Forehead height	−0.3	−2.05^*^ (0.05)	−83.61	−0.59	Eyebrow height	0.43	−2.86^**^ (0.01)	69.02	399.64
PD	−0.3	−2.18^*^ (0.04)	−1.19	−0.05	PD	−0.27	−2.23^*^ (0.03)	−1.25	−0.06
WHR	0.49	−3.41^**^ (0.00)	35.05	136.67	WHR	0.70	4.68^**^ (0.00)	85.31	214.47
*F*_(3, 42)_ = 5.50, Adjusted *R*^2^ = 0.23, *p* < 0.01^**^					*F*_(3, 42)_ = 11.36, Adjusted *R*^2^ = 0.41, *p* < 0.01^**^				

Regression method is backward elimination. Dependent variable is average Babyfacedness rating of each face. Significant differences are indicated:

* p < 0.05,

** p < 0.01 (two-tailed tests).

### Trait impressions of the babyface

Attractiveness and health are kinds of evolutionary traits and related more directly to evolutionary tendencies, so we analyzed them individually. We conducted a factor analysis on the other 15 traits. Bartlett's test of sphericity was significant, $\chi_{\left(66\right)}^{2}$ = 2028.45, *p* < 0.01, showing a factor structure, KMO = 0.896; these traits can be analyzed by factors. Principal component analysis and Promax rotation (Kappa = 4) were adopted. Items with the loading of < 0.60 were gradually deleted. One more analysis will be conducted with every change. Twelve remaining traits are presented in Table 6. Following the stereotype content model (Caprariello et al., 2009; Cuddy et al., 2009), these traits were divided into two categories—warmth and competence. Commonalities of all the items were more than 0.50; 67.44% of the total variance was explained by warmth and competence.

**Table 6:** **Component matrix: trait impressions of babyface**.

**Trait**	**Factor**	
	**Warmth**	**Competence**
Kindness	0.881	
Caring	0.854	
Threat	−0.838	
Honesty	0.805	
Aggressiveness	−0.800	
Trustworthiness	0.768	
Friendliness	0.763	
Likability	0.702	
Naïveté	0.702	
Leadership ability		0.807
Confidence		0.786
Intelligence		0.742

Kindness, caring, trustworthy, honesty, threatening, friendliness, likability, aggressiveness, and naïve are labeled as warmth traits; leadership, confidence, and intelligence are labeled as competence traits.

We conducted MANOVA to analyze attractiveness with the perceived age as covariates, culture (Two cultures:0 = Chinese, 1 = American), face gender (Two face genders:0 = Female, 1 = Male), and babyfacedness (Two babyfacedness levels:1 = Low, the least babyfaced face chosen in the second stage; 2 = High, the most babyfaced) as factors. There is no significant influence from perceived age [*F*_(1, 255)_ = 0.61, *p* = 0.44, η${}_{p}^{2}$ = 0.00]. Babyfacedness has a significant main effect [*F*_(1, 255)_ = 74.98, *p* < 0.01, η${}_{p}^{2}$ = 0.23].

**Figure 3:**
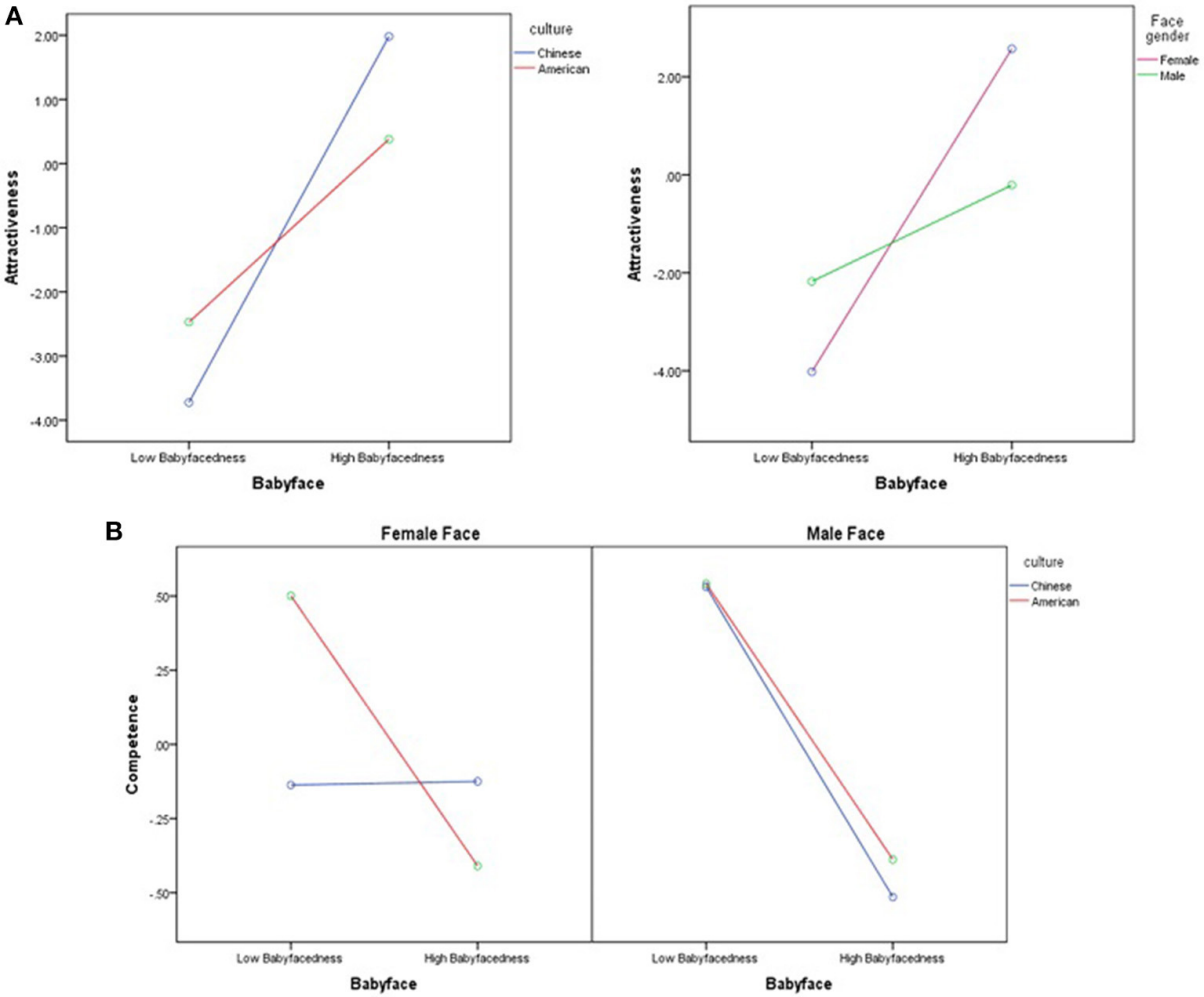
**Interaction effects among the variables of the babyface, face gender, and culture**. **(A)** Interaction effects between the babyface and culture, babyface and face gender on attractiveness. Covariate: perceived age = 31.54. **(B)** Interaction effects between the babyface and culture for two face genders on competence. Covariates: attractiveness = −0.96, perceived age = 31.54.

We analyzed the simple effect of significant interaction effects among the variables of babyfacedness, face gender, and culture. We found that when perceiving female [*F*_(1, 255)_ = 97.33, *p* < 0.01, η${}_{p}^{2}$ = 0.28] and male faces [*F*_(1, 255)_ = 10.03, *p* < 0.01, η${}_{p}^{2}$ = 0.04], regardless of whether Chinese [*F*_(1, 255)_ = 82.40, *p* < 0.01, η${}_{p}^{2}$ = 0.24] or American [*F*_(1, 255)_ = 18.78, *p* < 0.01, η${}_{p}^{2}$ = 0.07], the attractiveness of the high-level babyface is significantly higher than the low-level. But a high-level babyface seems to be more attractive [*F*_(1, 255)_ = 7.62, *p* < 0.01, η${}_{p}^{2}$ = 0.03] and a low-level babyface seems to be less attractive [*F*_(1, 255)_ = 4.59, *p* = 0.03, η${}_{p}^{2}$ = 0.02] for Chinese participants than Americans. A male mature face [*F*_(1, 255)_ = 9.95, *p* < 0.01, η${}_{p}^{2}$ = 0.04] and a female babyface [*F*_(1, 255)_ = 22.83, *p* < 0.01, η${}_{p}^{2}$ = 0.08] look more attractive (Figure 3A).

We conducted MANOVA to analyze health, warmth and competence traits with attractiveness and perceived age as covariates, because there is positive correlation between attractiveness, health, warm traits (Table 7), and babyfacedness and age effect on the babyface (Boshyan et al., 2014; Zebrowitz and Franklin, 2014). Attractiveness [Wilk's lambda = 0.74, *F*_(3, 252)_ = 30.19, *p* < 0.01, η${}_{p}^{2}$ = 0.26], perceived age [Wilk's lambda = 0.91, *F*_(3, 252)_ = 8.34, *p* < 0.01, η${}_{p}^{2}$ = 0.09] and babyfacedness [Wilk's lambda = 0.66, *F*_(3, 252)_ = 44.24, *p* < 0.01, η${}_{p}^{2}$ = 0.35] have significant influence when considered jointly on the variables health, warmth, and competence.

**Table 7:** **Means, standard deviations, and correlations between the variables: age, attractiveness, health, warmth, and competence**.

	**1**	**2**	**3**	**4**	**5**
(*N* = 264)					
1. Age	1				
2. Attractiveness	−0.33^**^, *p* < 0.01	1			
3. Health	−0.20^**^, *p* < 0.01	0.42^**^, *p* < 0.01	1		
4. Warmth	−0.28^**^, *p* < 0.01	0.60^**^, *p* < 0.01	0.48^**^, *p* < 0.01	1	
5. Competence	0.22^**^, *p* < 0.01	0.26^**^, *p* < 0.01	0.34^**^, *p* < 0.01	0, *p* = 1	1
*M*	31.54	−0.96	1.76	0	0
*SD*	7.12	4.27	3.40	1	1

Significant differences are indicated:

** p < 0.01 (two-tailed tests).

A separate ANCOVA was conducted for each dependent variable with attractiveness and perceived age as covariates. We analyzed simple effect of significant interaction effects among the variables of babyfacedness, face gender, and culture.

Attractiveness has a significant main effect on health rating [*F*_(1, 254)_ = 21.08, *p* < 0.01, η${}_{p}^{2}$ = 0.08], warmth [*F*_(1, 254)_ = 46.82, *p* < 0.01, η${}_{p}^{2}$ = 0.16], and competence [*F*_(1, 254)_ = 44.52, *p* < 0.01, η${}_{p}^{2}$ = 0.15]. Perceived age has a significant main effect on warmth [*F*_(1, 254)_ = 11.77, *p* < 0.01, η${}_{p}^{2}$ = 0.04] and competence [*F*_(1, 254)_ = 6.71, *p* < 0.01, η${}_{p}^{2}$ = 0.03], but not health [*F*_(1, 254)_ = 0.14, *p* = 0.71, η${}_{p}^{2}$ = 0.00]. Babyfacedness level also has a significant main effect on warmth [*F*_(1, 254)_ = 108.22, *p* < 0.01, η${}_{p}^{2}$ = 0.30] and competence [*F*_(1, 254)_ = 25.43, *p* < 0.01, η${}_{p}^{2}$ = 0.09], but not health [*F*_(1, 254)_ = 2.32, *p* = 0.13, η${}_{p}^{2}$ = 0.01].

The interaction effect of culture, face gender, and babyfacedness was significant, *F*_(1, 254)_ = 6.17, *p* < 0.01, η${}_{p}^{2}$ = 0.02. Figure 3B shows that the male babyface shows less competence than mature faces for both Chinese [*F*_(1, 254)_ = 22.10, *p* < 0.01, η${}_{p}^{2}$ = 0.08] and Americans [*F*_(1, 254)_ = 18.64, *p* < 0.01, η${}_{p}^{2}$ = 0.07]. However, cultural differences are found for female faces. The female babyface looks less competent for Americans [*F*_(1, 254)_ = 13.71, *p* < 0.01, η${}_{p}^{2}$ = 0.05], but no significant differences are found between babyface and mature faces for Chinese [*F*_(1, 254)_ = 0.00, *p* = 0.96, η${}_{p}^{2}$ = 0.00]. Compared with the Chinese, Americans are convinced that female mature faces are significantly more competent [*F*_(1, 254)_ = 8.75, *p* < 0.01, η${}_{p}^{2}$ = 0.03]. Gender differences were also found in that for Chinese judges, male mature faces showed more competence than female mature faces.

## Discussion

The babyface phenomenon seems to be easily found among male Chinese faces, which is similar to white faces (McArthur and Apatow, 1984; Berry and McArthur, 1985). However, we also found the babyface effect on female faces, which may be due to the earlier cessation of growth which causes female faces to retain more neotenous traits (Jones et al., 1995; Tanikawa et al., 2015). But because of the halo effect of attractiveness, further analysis is needed. In the second stage of our experiment, participants graded the babyfacedness at all babyface levels; we did not find significant gender differences when considering the effect of culture. Since we did not ask participants to evaluate the attractiveness of all faces, we may not be able to control attractiveness as a covariate when analyzing the relationship between facial features and babyfacedness. But, with the data of trait evaluation tasks in the third stage, we conducted an ANOVA, with attractiveness, culture and face gender as independent variables and babyfacedness as dependent variables. The main effect of attractiveness was significant, *F*_(14, 208)_ = 6.11, *p* < 0.01, η${}_{p}^{2}=0.29$. The interaction effect between attractiveness and face gender was not statistically significant, *F*_(14, 208)_ = 1.73, *p* = 0.052, η${}_{p}^{2}\;=\;0.10$. According to the result, we may not indicate that babyfacedness ratings of female faces co-vary by attractiveness more, because these data did not include all levels of babyfacedness. One possible explanation is that either male or female faces co-vary by attractiveness. More evidence is needed in the future.

We found that a lower forehead and closer PD were indices of the Chinese babyface. A different definition may be needed for the Chinese babyface. According to previous research about Caucasian faces, the babyface is usually defined as a round face with big eyes, wide PD, high raised eyebrows, a small nose, and low vertical placement of features, which yields a large forehead and a small chin. In our study, a Chinese babyface, a face with a high level of babyfacedness, has bigger eyes, a lower forehead, higher eyebrows, a smaller chin, a narrow PD, and greater WHR. The finding that faces with bigger eyes, higher eyebrows, a narrower chin and greater WHR were more babyfaced replicates many previous studies by Zebrowitz et al. (2015), including studies examining East Asian faces. But the facial features inferring the Chinese babyface, a lower forehead, and a narrower pupil, are new findings. Berry and McArthur (1985) and McArthur and Berry (1987) did not find that forehead height was related to American babyface. We can find that participants use different facial structures to determine the Chinese babyface and Caucasian babyface. Cunningham et al. (1995) demonstrated an evolutionary mechanism which can explain this difference. Cultural groups will modify their reproductive strategies. Comparing Whites and Blacks, greater sexual restraint is produced among Asians because of the harsh climate in North Asia. To get limited resources in limited time, evolutionary changes occurred in their appearance and life style. A round face shape, especially a babyface, will help them to delay and reduce their sexual activities. Asians may be faster on the evolutionary trend of babyface. Hence, people use different definitions to judge the Chinese babyface and Caucasian babyface.

What's more, we find interaction effects between culture and forehead height, eyebrow height, chin width, and WHR on the babyfacedness of Chinese faces. Chinese participants consider a face as more babyfaced with a lower forehead, higher raised eyebrow and greater WHR than American participants. But for Americans, a narrower chin contributes more for the babyfacedness of a face.

When evaluating the babyfacedness of a face, WHR and PD contribute the same to babyface perception for Chinese and American judges. But, cultural differences show that Chinese judges care more about forehead height, because the Chinese are concerned more with the combined result of all facial features. Forehead height is related to the entire facial shape, accounting for a high proportion of a face. Peng and Nisbett (1999) and Nisbett et al. (2001) indicated that it is the result of a holistic cognitive style. In contrast, Americans are accustomed to analytical thinking. One or a few prominent characteristics, such as eyebrow height, will lead to a conclusion of babyface. This finding verified our hypothesis. Different cultural groups with different cognitive styles will take different facial features into account.

**Figure 4:**
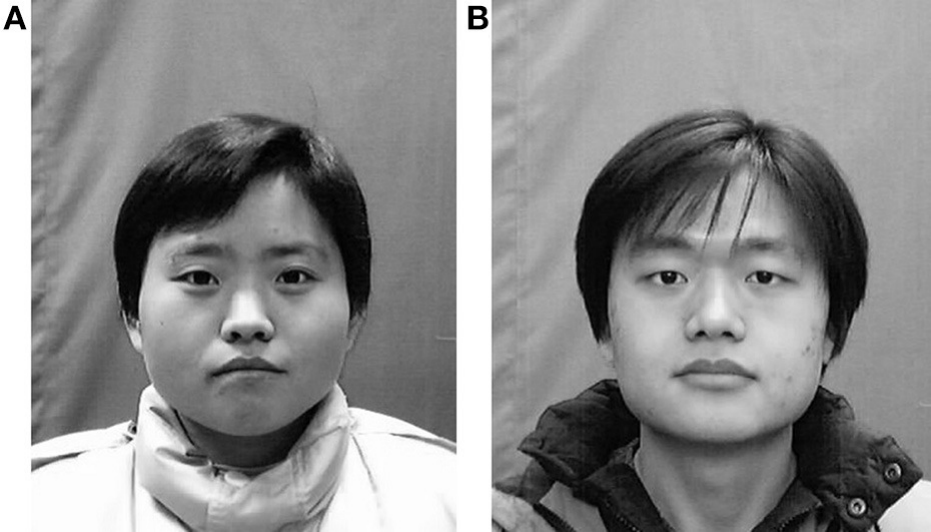
**A pair of typical Chinese babyfaces, female babyface, (A) and male babyface (B)**.

Based on the coefficients of the mixed model, the influence of culture has a significant tendency (*p* = 0.08). When judging on a same Chinese face, especially a babyface, American tend to rate it higher in babyfacedness. A Chinese babyface (Figure 4) is usually perceived to be more babyfaced for the American judges than for the Chinese judges. This result is inconsistent with evidence that Korean faces are judged more babyfaced than White faces by both American and Korean judges, and Korean judges rate both Korean and White as more babyfaced than do American judges (Zebrowitz et al., 2007a). Both Chinese faces and Korean faces are kinds of Asian faces. But these two kinds of faces are not totally the same. Previous studies show DNA evidence of Koreans and we have reasons to infer that the shape of Korean's eyes is more narrow and elongated (Jin et al., 2003, 2009). But as we know, bigger eyes are the key index of a babyface. From this perspective, Chinese faces seem to be more babyfaced than Korean faces and also American faces. Zebrowitz et al. (2007a) didn't indicate if the rates of Korean and American differ systematically. Their explanation is that it comes from face race effect. We found that a typical Chinese babyface is judged more babyfaced by Americans than Chinese. One possible reason is that Chinese faces are more babyfaced than Korean faces and American faces. Even with the effect of face race, Americans still judge Chinese babyfaces as more babyfaced. More research is needed.

We argue that a babyface is more attractive in general, which is consistent with the babyface overgeneralization hypothesis and the halo effect of attractiveness. There is a remarkable agreement in the warmth inference of the babyface all over the world (Zebrowitz et al., 2012), which is also true in our study. However, an interesting cultural difference is that Chinese participants seem to be more extreme. They consider a high-level babyface as more attractive and a low-level babyface as less attractive than Americans. Apparently, Chinese people like the female babyfaces and male mature faces more, which can be easily explained by evolutionary tendencies (Buss, 1989; Zebrowitz, 2003; Zebrowitz and Montepare, 2008). This phenomenon may also reflect a preference for own-race faces, which is consisted with previous research (Zebrowitz et al., 2007a).

It is usually believed that the babyface can lead to impressions of weakness, obedience, naïve characteristics (McArthur and Apatow, 1984; Berry and McArthur, 1985) and more femininity (Buss, 1989; Zebrowitz, 2006). Zebrowitz et al. (2012) only examined male faces and found a negative effect of babyface on health. Zebrowitz and Franklin (2014) tested both male and female faces and found a stronger influence of babyface on older adults than young adults in the impressions of health. However, we found no effect of babyface on health rating among young adults. Because the halo effect of attractiveness makes faces look healthier, we only found that attractiveness has a significant effect on health.

In our study, we found that for male Chinese faces, both Chinese and Americans believed that the babyface shows less competence than mature faces. But for the female Chinese faces, the Chinese do not consider the female babyface as less competent, but it is less competent for American judges. The American inference about Chinese female babyfaces may simply be a natural extension of the babyface in general. However, given that the babyface is an evolutionary tendency and survival strategy, a Chinese female babyface implies more fertility and attractiveness, but no less competence. Because it is an evolutionary result, there is no doubt that a female babyface shows the same competence with mature faces for Chinese participants. Furthermore, Chinese may have often encountered numerous competent Chinese women who happen to have babyfaces; naturalistic realism may be the core source of the cultural differences. Additional research is needed to evaluate if babyfaced Chinese women also have the same social status and power as their peers. Gender differences suggesting that mature male faces are more competent than mature female faces for the Chinese can be explained by evolutionary tendencies (Buss, 1989; Zebrowitz, 2003; Zebrowitz and Montepare, 2008). A man with a mature face may possess more resources, more wealth, and higher social status.

The current study suggests that the facial structures and first impressions of the babyface are not necessarily universal. A Chinese babyface may be more babyfaced in the eyes of Americans, but it is not perceived to be less competent, and may be seen as even more attractive in the eyes of the Chinese. Recognizing the cultural differences in an evolution-based natural phenomenon may enrich our understanding of human commonality and diversity.

## Author contributions

WZ developed the study concept with KP. WZ developed the experimental paradigm with QY and FY. WZ and QY conducted the experiment and collected the data. WZ performed the data analysis and interpretation under the supervision of KP. WZ and KP drafted the manuscript. All authors contributed to the discussion of the manuscript. QY and FY provided critical revisions. All authors approved the work for publication.

## Notes

1. According to Berry and McArthur (1985), forehead height_1_ is the distance between highest point of forehead and the connection of the two highest eyebrow points.
2. According to Zebrowitz-McArthur and Montepare (1989), forehead height_2_ is the distance between highest point of forehead and the connection of two pupil center points.

## Funding

This work was supported by National Nature Science Foundations of China (No. 31170973 and No. 31471001) and Tsinghua University Top Genius Training Program (Spark Plan).

### Conflict of interest statement

The authors declare that the research was conducted in the absence of any commercial or financial relationships that could be construed as a potential conflict of interest.
